# Cognitive behavior therapy in the treatment of panic disorder

**DOI:** 10.4103/0019-5545.49450

**Published:** 2009

**Authors:** M. Manjula, V. Kumariah, P. S. D. V. Prasadarao, R. Raguram

**Affiliations:** Department of Mental Health and Social Psychology, National Institute of Mental Health and Neurosciences, Bangalore - 560029, India; 1 Department of Psychiatry, Kempegowda Institute of Medical Sciences, Bangalore - 500 004, India

**Keywords:** Clinically significant change, magnitude of change, multicomponent cognitive behavioral intervention

## Abstract

**Background:**

Comprehensive cognitive behavior therapies have been proved to be more effective than behavioral interventions. However, the efficacy of CBT is not studied in the Indian context and also, the amount of change brought about by CBT is not known. Aims: This study aims to examine the efficacy of cognitive behavioral intervention (CBI) in the treatment of panic disorder. Our specific objectives were to assess the effectiveness of CBI in reducing symptom severity as well as cognitions related to panic and panic-related behaviors. Design: The study adopted a two-group comparison with pre- and postassessments design.

**Materials and Methods:**

The sample consisted of 30 patients sequentially allotted to the CBI (n = 15) and behavioral intervention (BI, n = 15) groups. Assessment was done using a semistructured interview schedule, panic disorder severity scale, Texas panic attack record form, Anxiety Sensitivity Index, Agoraphobic cognitions questionnaire, Behavioral avoidance checklist, and Panic appraisal inventory. The CBI group was provided with comprehensive cognitive behavior therapy and the BI group with psycho-education and applied relaxation.

**Results:**

CBI was found to be superior to BI in the reduction of panic symptoms, behavioral avoidance, safety behaviors, and cognitions. A large percentage of the CBI group patients met the criteria for clinically significant change with a large magnitude of change.

**Conclusion:**

Multicomponent CBI is superior to BI in terms of the amount of change it brings about with respect to panic symptoms, avoidance, safety behaviors, and cognitions.

## INTRODUCTION

As evidenced by several trials, Cognitive Behavior Therapy (CBT) is a highly effective treatment for panic disorder. The outcome of CBT is comparable to that of pharmacotherapy with continued use of medication at posttreatment being found to result in poorer outcomes for CBT.[1,2] The results of studies on manualized CBT suggest that a substantial proportion of patients with panic improve and remain thus with up to 85% of the patients found to respond positively to an average of 10-15 sessions of CBT.[3] Various multicomponent therapies based on the cognitive behavioral paradigm, which include techniques to lower physiological arousal (relaxation training), reduce cognitive misinterpretation (cognitive restructuring), develop coping skills, breathing retraining, interoceptive exposure, and exposure to feared stimuli are found to be effective compared to single-component therapies.[4] CBT has been found to be more cost-effective over the long term (two years) than pharmacotherapy.[5] It has shown promising results in challenging problems associated with the discontinuation of benzodiazepines, antidepressants, and SSRIs.[6,7] It is also found to be effective in the treatment of drug-resistant panic disorder.[8]

Various studies have been conducted comparing different cognitive behavioral techniques in the treatment of panic disorder; a couple of studies conducted on Applied Relaxation (AR) show that these techniques seem to be equally effective as comprehensive CBT. In an early study comparing AR with *in vivo* exposure; AR was found to be as effective as exposure at posttreatment and at 15-month follow-up, with 65% of the subjects achieving clinically significant improvement.[9] A subsequent study examining the efficacy of AR and progressive muscle relaxation (PR) found AR to be superior in reducing the symptoms, anxiety, and maintenance of gains compared to PR at one-year follow-up.[10] Comparison of AR with *in vivo* exposure and cognitive methods with instructions for self-exposure yielded significant improvements for all three treatments that were maintained at one-year follow-up. However, clinically significant improvement was achieved by a greater number of patients who received AR, both at posttreatment and at follow-up.[11] Although the above studies show that the effect of exposure is built into AR, the studies which have the components of interoceptive exposure and exposure show that dealing specifically with avoidance, escape and safety-seeking behaviors does not happen unless specifically addressed.[12] There is considerable and strong evidence to show that the CBT packages that include interoceptive exposure are efficacious for panic in comparison to relaxation. Relaxation is said to result in greater reductions in generalized anxiety associated with panic attacks but has been associated with high dropout rates. The percentage of people reporting a panic-free state varied significantly among CT (65-90%) and RT (47-65%) therapies.[13]

Information with respect to the different components of CBT and its impact on the different symptoms is too limited to add any conclusive information. One such study[14] compared CT plus *in vivo* exposure and associative therapy (AT) plus *in vivo* exposure. Initial CT produced a significant reduction in panic frequency, whereas AT did not affect panic. Only after the addition of exposure was there a clear reduction in depression, state or trait anxiety, self-rated agoraphobia, and behavioral avoidance. These findings highlighted the importance of CT in reducing panic and exposure in antiagoraphobia.[14] Long-term treatment (3-9 yrs) of *in vivo* exposure in patients with agoraphobia, with or without panic disorder, showed 40% achieving complete remission, 36% with mild-to-moderate agoraphobia, and 24% who were nonresponders and suffered from severe agoraphobia at follow-up.[15]

Most of the studies on CBT in panic disorder have compared different combinations of techniques rather than using multicomponent therapies. The studies have not used any comprehensive assessment of the different components of panic. Multiple co-morbid conditions were included in the studies and behavioral components such as avoidance and safety behaviors have not been assessed properly. Although Applied Relaxation is found to be effective in the treatment of panic, most studies did not include this as a component of the therapy and this lacuna has been pointed out in APA guidelines for panic disorder as well.[16] Studies comparing the CBT and relaxation did not control for exposure. As instructions are said to play a significant role in self-exposure, not controlling for this factor would become a confounding variable in itself.[11] One study comparing cognitive therapy and relaxation did control for this, but did not use comprehensive assessments.[17]

The present study is an attempt to use comprehensive CBT which includes different components of CBT that are found to be effective in the treatment of panic in a holistic manner in a short duration of five weeks. The group treated with applied relaxation (AR) was taken as a control group and was not asked to refrain from self-exposure. These controls were asked to use AR as a coping technique during panic attacks in order to see the maximum impact of this technique. This study also intends to examine the effectiveness of cognitive and exposure techniques in addition to a relaxation technique and is one of the first in its kind carried out in the Indian setting. The aim of this study was to study the efficacy of Cognitive Behavioral Intervention (CBI) in patients with panic disorder. The specific objectives were to study the efficacy of CBI in reducing symptom severity, cognitions, and panic-related behaviors among individuals with panic disorder.

## MATERIALS AND METHODS

### Sample

The sample comprised of 15 individuals each in the Cognitive Behavioral Intervention (CBI) and Behavioral Intervention (BI) groups. The study adopted a two-group between- and within-group comparison design. The sample was taken from the inpatient and outpatient Departments of Psychiatry from two reputed hospitals. One was a tertiary psychiatric hospital whereas the other was a psychiatry department in a general hospital. Most of the cases (26/30) were taken from the tertiary psychiatric hospital. The sample had to fulfill the following inclusion criteria: The ICD-10 Diagnostic Criteria for Research[18] for panic disorder with or without agoraphobia (F 41.00, 40.01), with or without depression (F 32.0, 32.01) or dysthymia (F 34.1), and Current panic disorder severity of at least 2 on a Panic Disorder Symptom Severity Scale;[19] in the age range of 18-55 years, and who could read, write, and understand English or the local language. The exclusion criteria were clinical history suggestive of psychotic disorders, psychoactive substance abuse disorders, hypertension or hypothyroidism, other co-morbid conditions, and prior exposure to structured psychological intervention for panic disorder.

### Measures

A semi-structured interview schedule was developed to obtain sociodemographic and clinical details of the sample. The severity of the panic disorder was assessed by using the Panic Disorder Severity Scale (PDSS).[19] This is a seven-item clinical interview assessment instrument that rates core features of panic disorder. The items include frequency of panic attacks, distress caused by panic, anticipatory anxiety, agoraphobic fear/avoidance, and work and social impairment. Each item is rated on a 0-4 scale, where 0 indicates “not present” whereas 4 is considered “extreme, pervasive, and disabling.” The frequency and other details of the panic were assessed using the Texas Panic Attack Record form,[20] which is a structured diary maintained by patients. For each panic episode, the individual is required to record the date, time, duration, severity of symptoms and setting parameters (*e.g*., place, activity etc), bodily sensation, thoughts, and the ways in which the panic attack was handled. The Anxiety Sensitivity Index (ASI)[21] was used to measure dispositional tendencies to misinterpret bodily sensations, which arise from the belief that these sensations have harmful somatic, psychological, and social consequences. The scale has 16 items rated on a 0-4 scale, where 0 indicates ‘very little’ and 4 ‘very much’. The catastrophic cognitions related to panic were assessed using the Agoraphobic Cognitions Questionnaire (ACQ)[22] consisting of 14 catastrophic thoughts rated on a five-point scale ranging from “not frightened or worried” to “extremely frightened” by sensations.

The dimension of cognitive appraisal of panic was assessed by using the Panic Appraisal Inventory (PAI)[23] which assesses the following dimensions: 1) anticipated panic; 2) panic consequences, and 3) panic coping. The scale has 45 items rated on a 0 (no chance)-100 (definite panic occurrence) scale. A check list (Behavioral Avoidance Checklist, BAC) was developed to assess panic-related phobias which included panic symptoms and bodily sensations, social, specific, and agoraphobic situations, and safety behaviors used in these situations. It has two sections: a) agoraphobic avoidance with 34 items, and b) Safety Behaviors with 16 items. The items are rated on a five-point scale where 1 is never and 5 always. The items of the checklist were validated by two senior clinicians and were also tried out in the pilot phase to make the necessary changes.

### Procedure

The patients fulfilling the specified inclusion and exclusion criteria and stabilized on medication (two months of adequate trial of the drugs with proven efficacy) or drug naïve patients were evaluated clinically and referred by the psychiatrists. These patients were interviewed for their suitability and willingness to participate in the study; treatment conditions were explained and informed consent was sought. The patients thus selected were sequentially allotted to two groups. A total of 314 patients were screened out of which 115 did not meet the severity criteria. Fifty-eight refused because of the difficulties in coming for therapy, 18 did not give consent, six had undergone therapy, 12 consented but did not come, 35 did not meet the criteria for age, co-morbidity etc, 13 dropped out, 20 deferred therapy, seven were not stabilized and there were changes in medicines, but 30 finally completed the therapy. The therapy and the assessments (both pre- and post-) were carried out by the first author for both the groups as a part of her Ph.D. thesis work. The ethics committee and protocol review committee of NIMHANS approved the thesis protocol. The CBI group received a comprehensive multicomponent cognitive behavioral intervention program that included the following components: psycho-education, applied relaxation, cognitive restructuring, interoceptive exposure, and *in vivo* exposure. Patients in the BI group received psycho-education and applied relaxation.

### The therapy and assessments for CBI and BI groups

Assessments were carried out at the beginning of the therapy (pretreatment) and subsequently at the end of every week for both the groups; totally six assessments were carried out in five weeks. However, only the results of the pre- and postassessment scores are presented in this paper. For the CBI group, psycho-education and applied relaxation were given in the first week, AR was continued in the second week, cognitive restructuring was done in the third week, interoceptive exposure was done in the fourth week, and *in vivo* exposure in the fifth week. The total number of sessions ranged from 15 to 20, spread over five weeks.

For the BI group, psycho-education and applied relaxation training was given for 8-10 sessions spread over two weeks. In the next three weeks, patients were seen once in a week for weekly assessments and instructed to continue applied relaxation.

### Analysis of data

The analysis was carried out using Statistical Package for Social Sciences 10.0 version for windows (SPSS 10.0). Statistical techniques included quantitative and qualitative analysis: t-tests were used to compare the means of the CBI and BI groups for the significance of the differences of the outcome variables. The ‘chi-square’ tests were used to compare the groups for categorical variables such as sex, education etc., on the SSIS and the items in the panic diary. Paired ‘t’ tests were used to compare the within group differences from pre- to postassessments.

Effect sizes (ESs) were calculated to find out the effect of change in two treatment conditions on the outcome variables. Cohen's[24] classification scheme was used to evaluate the magnitude of ES. Both within-group and between group estimates of effect sizes were calculated for each outcome measure. Unbiased estimates of ES were calculated to correct for the small sample size.[25]

### Clinically significant improvement

Clinical significance describes the meaningfulness that a treatment produces benefits from the perspective of a clinician or the patient.[26] Clinically meaningful change brings a person to within normal limits in relation to a normative population. Clinically significant improvement was assessed using Jacobson and Truax's[27] method.

The first step is to establish a cutoff point for each client that must be crossed in moving from the dysfunctional to the functional distribution (postassessment scores within 2 SD of pretherapy scores added in the direction of functionality).

A second step is to determine the reliability of the change from pre- to posttest. Each client was assessed on the Reliable Change Index (RCI).[28] An RCI score of ≥ 1.96 is reflective of clinically significant change. Based on these two criteria, clients are classified as “recovered” if they passed cutoff and RCI criteria, “improved” if they passed RCI criteria independent of whether they were in the functional range, “functional” if they met cutoff scores alone, ‘’improved but not recovered” if they showed reliable improvement but continued to be in the dysfunctional range after therapy, and “unchanged” if they did not pass either of the criteria.

## RESULTS

The demographic and clinical details are given in Table 1. The mean age of the CBI group was 30.80 years (± 9.14) and that of the BI group was 36 years (± 10.71). The majority of the patients in both the groups were males and about half of the samples in both groups were graduates. The majority of the subjects (86.7% in both the groups) were Hindu and the remaining subjects were of other faiths (Christians and Muslims). There were no significant differences in the demographic variables between the groups, except for the background.

**Table 1:** Table 1: Demographic and clinical details of the two groups

		CBI group (*n* = 15)		BI group (*n* = 15)		
				
		*n*	%	*n*	%	*x*^2^value
Age (in years)	21-30	9	60	6	40	1.89 NS (df = 2)
	31-40	4	26.7	4	26.7	
	41-55	2	13.3	5	33.3	
Sex	Male	12	80	10	66.7	0.68 NS (df = 1)
	Female	3	20	5	33.3	
Education	High school and pre-university	7	46.7	5	33.3	1.33 NS (df = 1)
	Graduation	7	46.7	7	46.7	
Income (per month)	Below Rs. 3000	7	46.7	3	20	2.43 NS (df = 2)
	Above Rs. 3000	5	33.3	7	46.7	
	Above Rs. 10,000	3	20	5	33.3	
Background	Urban	9	60	15	100	7.50^*^
	Rural	6	40	0	0	
Marital status	Married	9	60	5	33.3	2.14 NS (df = 1)
	Unmarried	6	40	10	66.7	
Diagnosis	Panic disorder (PD)	10	66.7	13	86.7	1.73 NS (df = 2)
	PD with agoraphobia	3	20	1	6.7	
	PD with dysthymia	2	13.3	1	6.7	
Situations triggering panic	Fear evoking	1	6.7	1	6.7	0.62 NS (df = 2)
	Without any trigger	10	66.7	8	53.3	
	Under both	4	26.7	6	40	
Frequency (in the past month)	Daily	6	40	9	60	2.27 NS (df = 2)
	> Twice/week	8	53.3	4	26.7	
	> Thrice/month	1	6.7	2	13.3	
Treatment details	No treatment	7	46.7	9	60	3.36 NS (df = 3)
	SSRI + BZP	4	26.7	5	33.3	
	TCA + BZP	1	6.7	1	6.7	
	SSRI + TCA + BZP	3	20	0	0	
Improvement	Not at all	8	53.3	8	53.3	0.27 NS (df = 2)
	25%	4	26.7	3	20	
	50%	3	20	4	26.7	

Panic disorder was the major and the only diagnosis in most of the patients in the two groups, displaying a continuous course, spontaneously occurring panic attacks, and with a daily frequency of panic attacks in about half of the sample [Table 2]. The mean number of attacks reported in the week prior to coming for therapy was 5.86 (± 4.92) and 7.80 (± 6.28) in the CBI and BI groups respectively, indicating that they had attacks almost every day. Appetitive behaviors such as drinking water, juice, eating, taking rest, and distractions were reported as being useful in reducing the number of attacks (10 and 13 in the CBI and BI groups respectively). The duration of illness was 33.20 (± 69.84) and 30.68 (± 33.02) weeks in the CBI and BI groups respectively; the duration of medication was 22.20 (± 61.20) and 16.46 (± 29.90) weeks, indicating that the subjects were on a stable medication (as confirmed by the treating psychiatrists) for at least four months before entering into therapy. The details of the places, situations, and activities avoided by the patients in the CBI and BI groups are as follows: Traveling and riding: 26.7% each; staying alone or going out alone: 53 and 26.7%; closed places like theatres, queues, class rooms etc.: 13% each; hard work: 33.3 and 60%; stressful situations like fights, arguments, funerals, sick people: 53.3 and 13.3%; dietary restrictions: 20 and 13.3%; noise, talking aloud: 26.7% each; attending to work requiring responsibility: 60 and 26.7%. The avoidance noticed in the samples is not typical of agoraphobic avoidance; this may be an artifact of majority of the group having panic disorder alone as the diagnosis and very few cases with agoraphobia of mild to moderate severity. There was no significant difference between the two groups with regard to any of the clinical variables and both the groups were comparable on the outcome variables.

**Table 2:** Means, SDs, and t-values of pre- and postassessment scores of the two groups on outcome measures

	CBI group (*n* = 15)					BI group (*n* = 15)				
	
Measures	pre		post			pre		post		
					‘t’ value					‘t’ value
	Mean	SD	Mean	SD		Mean	SD	Mean	SD	
No of attacks	4.66	4.95	1.00	1.25	2.59*	3.93	2.12	2.40	2.87	2.62
Duration	48.07	62.02	13.68	30.95	3.70^a^	66.40	94.33	19.44	35.39	1.95
Severity	5.72	1.87	1.30	1.68	6.22^a^	4.71	1.60	1.42	1.49	5.42^a^
Symptoms	5.53	1.24	1.13	1.35	3.67**	4.26	1.38	1.60	1.80	1.16
PDSS (Total)	3.10	0.51	0.48	0.42	17.42^a^	3.28	0.48	1.30	0.84	8.54^a^
ACQ	35.93	7.75	16.33	3.53	13.31^a^	34.73	10.14	21.40	8.78	4.62^a^
ASI	32.66	11.31	7.40	9.43	9.21^a^	36.20	10.51	22.33	14.19	3.70**
BACA	67.52	25.12	41.80	14.47	4.22^a^	62.26	27.90	51.73	28.23	3.05**
BACSB	41.60	11.78	18.66	5.13	7.48^a^	39.20	11.88	29.80	13.85	2.82**
PAIA	22.40	22.39	8.19	15.61	3.30**	20.80	20.86	12.54	20.17	2.55*
PAIP	5.38	3.82	0.36	0.77	5.53^a^	4.12	2.13	1.08	1.37	5.54^a^
PAIS	3.90	3.22	0.37	1.03	4.54^a^	3.33	2.73	1.22	2.43	2.80**
PAIM	2.96	2.54	0.22	0.65	4.03^a^	2.04	2.75	0.88	2.01	1.70NS
PC	22.96	18.56	76.40	18.16	9.00^a^	25.78	25.35	58.66	28.30	4.14^a^

ASI: Anxiety sensitivity index; ACQ: Agoraphobic cognitions questionnaire; PAIA: Panic appraisal inventory anxiety subscale; PAIP: Physical consequences subscale; PAIS: Social consequences; PAIM: Mental consequences; PC: Panic control, BACA: Behavioral avoidance checklist anxiety subscale; BACSB: Safety behaviors subscale.

* *P* < 0.05

** *P* < 0.01

a *P* < 0.001, df = 14

### Symptom severity of panic

To examine the efficacy of the intervention on symptom severity, the data were analyzed on Panic Disorder Severity Scale (PDSS) and Panic Diary (PD). Both the CBI and BI groups showed a significant difference between pre- and postassessments on all subscales of the PDSS. However, there is a tendency for lower post assessment scores in the CBI group.

Comparison of the postassessment scores of the two groups showed a significant difference on all the subscales and on the composite scores (t = 3.37, *P* < 0.001), showing A greater amount of change in the CBI group. The within group estimate of ES for the composite score on PDSS is 5.14 for the CBI and 4.12 for BI groups. On various subscales, the ESs for the CBI and BI groups were: 5.31 and 4.76 for frequency; 7.73 and 3.54 for distress, 3.04 and 2 for the anticipatory anxiety; 3.11 and 1.63 for the avoidance; 3.11 and 1.61 for fear of bodily sensations; 3.52 and 1.32 for impairment in work, and 2.55 and 1.81 for social interactions, respectively. Between-group ES was 1.21 showing a larger amount of change with CBI.

Comparison of panic attacks from the first to the last week within the two groups showed that the CBI group showed a significant reduction in all parameters whereas the BI group showed a reduction only in the severity of panic. However, a comparison of the postassessment scores of the two groups did not show any significant difference although there is a tendency for lower scores in the CBI group for all parameters.

### Panic-related cognitions

The anxiety sensitivity index (ASI) which is said to be a dispositional trait, was found to be reduced significantly in both the groups. However, there was a greater level of change with CBI, which is reflected in the significant difference in the postassessment scores between the two groups (t = 3.39, *P* < 0.001). The within group effect sizes for ASI were 2.53 and 1.23 for the CBI and BI groups, and the between-group ES was 1.24. In agoraphobic cognitions (ACQ), both the groups showed significant change from pre- to postassessment. However, comparison of post- scores showed a significant difference in the change brought about by the two interventions (t = 2.07, *P* < 0.05). Within-group ES was 2.53 and 1.31 for the CBI and BI groups respectively and the between group values was 0.76.

Similarly, on panic appraisal, the CBI group showed significant change on all the subscales whereas the BI group showed significant change on all scales except for the PAIM subscale. The comparison of post- scores indicated significant difference on the panic control subscale (t = 2.04, *P* < 0.05), indicating a greater confidence in controlling panic in the CBI group. The within group ESs for the different subscales of PAI are as follows: PAIA: 0.63 and 0.4; PAIP: 1.31 and 1.43; PAIS: 1.1 and 0.77; PAIM: 1.08 and 0.42; PC: 2.92 and 1.3 for the two groups. The between group ESs were 0.24, 0.65, 0.45, 0.44, and 0.73 for PAIA, PAIP, PAIS, PAIM, and PC respectively.

### Panic-related behaviors

Panic-related behaviors were assessed using the Behavioral Avoidance Checklist (BAC). Both groups showed a significant change from pre- to postassessment in the avoidance (BACA) and the safety behaviors (BACSB) [Table 2]. Comparisons of postassessment scores of the two groups showed that the difference on the avoidance scale was not significant (t = 1.21 NS) whereas the CBI group differed significantly from the BI group (t = 2.92, *P* < 0.01) in safety behaviors. Within-group estimates of ESs in the CBI and BI groups for behavioral avoidance were 0.96 and 0.36 respectively and for safety behaviors, they were 1.85 and 0.75. Between groups ES on the behavioral avoidance subscale was 0.44 (low ES) and it was 1.08 (large ES) for safety behaviors.

### Clinical significance and reliability of change

The cutoff scores considered to determine clinically significant change (CSC) for different scales are as follows: PDSS: 1; ASI: 12.67; ACQ: 17.55; PAIA: 5; for PAIP, PAIS, and PAIM, it is 0; PC: 75-100; BACA: 38.67, and BACSB: 16.96. Those scoring less than the cutoff or within the range were considered to be within the functional range. The patterns of CSC seen for the outcome measures in the two groups are presented in Table 3.

**Table 3:** Percentage of clients showing clinically significant change on the outcome measures in the two groups

Variables	1 Improved (%)		2 Functional range (%)		3 Recovered (Clinically significant change) (%)		4 Improved but not recovered (%)		5 No change (%)	
					
	CBI	BI	CBI	BI	CBI	BI	CBI	BI	CBI	BI
Composite score on PDSS	100	100	86.7	40	86.7	40	13.3	60	0	0
ASI	93.3	60	73.3	13.3	73.3	13.3	20	46.6	6.6	13.3
ACQ	100	73.3	73.3	53.3	73.3	53.3	26.7	26.7	6.6	26.7
PAIA	46.6	40	73.3	53.3	33.3	20	20	20	6.6	26.7
PAIP	73.3	60	80	40	53.3	46.6	20	33.3	0	33.3
PAIS	60	40	80	60	53.3	26.7	6.6	13.3	6.6	13.3
PAIM	53.3	6.6	86.6	80	40	6.6	6.6	0	0	6.6
PC	100	60	66.6	26.7	66.6	33.3	33.3	26.7	0	40
BACA	66.6	33.3	73.3	53.3	53.3	20	13.3	6.6	6.6	33.3
BACSB	93.3	33.3	66.6	26.7	60	20	33.3	13.3	6.6	40

A large percentage of the patients in the CBI group showed clinically significant change. The percentage of patients showing reliable change and falling in the functional range is also high in the CBI group. It is also evident that a higher percentage of patients in the BI group did not show any change. It can be noted that although this difference was not evident on the ‘t’ test and both the groups showed significant change in all the variables of panic severity, clinical significance methods provide a more accurate percentage of the patients showing recovery, thus, giving a clearer picture of clinical improvement in these patients.

## DISCUSSION

This study aimed at examining the efficacy of CBI in reducing panic symptoms and changing panic-related cognitions and behaviors. With respect to the panic symptoms, comparison of pre- to postassessment scores within the groups and post- scores between the groups on PDSS show that although both can bring about significant changes, there is a significant change in the CBI group as compared to the BI group. Also, the CBI group showed a significant difference on more variables on the Panic Diary than the BI group. These findings indicate that the extent of change that has taken place in the two groups was not the same. This is further supported by larger between-group ESs on PDSS for all variables and a composite score of 1.21, indicating a high magnitude of change in the CBI group compared to the BI group. The magnitude of the treatment effects was higher in the present study compared to other studies.[29,30]

The examination of CSC further gives a clear picture of the percentage of patients showing a reliable and functional change, which is consistent with the above findings. The fact that some patients in the BI group did not show significant improvement and some deteriorated indicates that behavioral techniques alone may not give significant change and there may be stagnation and reversal of the improvement if it is not followed by structured cognitive techniques to maintain the improvement.[31] A clinically significant change (recovered) was achieved in panic symptoms (PDSS) by 86.7 (80-100%) and 40% (33-67%) of the patients in the CBI and the BI groups respectively. Similar percentages (85-100%) for CBT in comparison to RT were reported in other studies.[30,32–34] However, a meta-analytical study reported the weighted proportion of CSC of 72% for CBT and 50% for RT.[13] One study reported a contradictory finding that applied relaxation and CBT were equally effective.[35] The reasons for the discrepancy may be that their relaxation treatment included exposure to bodily sensations among the anxiety hierarchies, inducing the effect of exposure.

The findings in the cognitive domain clearly indicate that the changes taking place by psycho-education and relaxation are not equivalent to that of dealing with cognitions in a structured manner. Similar findings have been reported by Dannon *et al*,[31] who found that the use of an information booklet was useful in decreasing anxiety and panic attacks in the first few weeks but that the effect disappeared with time. This may be because information alone may not be sufficient to change cognitive distortions and schemas which tend to be based on idiosyncratic evidences of each patient, which have to be specifically addressed.[12]

With respect to the appraisal of various consequences of panic also, it is clear that concerns of physical threat improved equally in both the groups. However, fear of mental consequences did not improve with BI, which may be because relaxation and education alone do not provide convincing evidence to change cognitions.[36] Thus, it can be understood that the significant change noticed in controlling panic can be attributed to the change in appraisal of mental consequences. The above findings clearly indicate that as expectation of panic has an important role in maintaining panic, cognitive intervention brings about a decrease in panic and maintenance of improvement due to a reduction in this expectation.[31,37] Similarly, there was a significant change in anxiety sensitivity with a large difference between the two groups, supporting the fact that even a dispositional tendency like anxiety sensitivity can change with cognitive intervention.[38]

For behaviors related to panic, significant reduction in only safety-seeking behaviors at postassessment between the groups may be an artifact of less severe avoidance in the sample as a whole. These findings are further supported by the data from the panic diary. At the end of five weeks, BI group subjects continued their safety behaviors such as going to the doctor, avoiding work, escaping from situations, lying down, drinking water/juice, eating etc. whereas the CBI group faced their situations, tried to relax, and used self instructions. It is consistent with the findings that safety behaviors help in the maintenance of the catastrophic cognitions.[39] Also, cognitive coping strategies such as trying to cope with the situation and achieving the goal as well as emotional coping strategies such as planned problem solving, confrontative coping, distancing, and positive reappraisal are considered as healthy coping patterns.[40]

As evidenced in the literature, unless specific instructions for stopping safety behaviors are provided, patients with panic continue such safety-seeking behaviors.[41] In the present study, the CBI group received cognitive restructuring which elaborated on safety behaviors and specific instructions not to involve in them, thus making a difference in these behaviors and the coping patterns. Interoceptive exposure to bodily sensations and *in vivo* exposure to the feared stimuli also would have helped them overcome avoidance and safety behaviors. These findings strengthen the fact that cognitive restructuring and the exposure play a significant role in the treatment of panic disorder. Multicomponent therapies have been found to be more useful because nearly all panic disorder patients are known to show at least some patterns of avoidance behavior, gross or subtle.[42]

The BI group continued to have panic attacks in the presence of triggering events like bodily sensations when alone, traveling, hard work, arguments and thoughts about panic along with spontaneous attacks, even at the end of six weeks. On the other hand, the CBI group did not panic in these situations any more. Similarly, the extent of change (ESs) and CSC achieved in the two groups indicates the importance of the cognitive change in reducing avoidance and safety behaviors and developing healthy ways of coping, thus contributing to the overall change in symptomatology.

Some of the safety behaviors used in this sample seem to be unique to our cultural background. These can be grouped as: 1) Appetitive behaviors: This included consuming specific foods based on the assumption that panic is due to the lack of energy (taking carbohydrate- and protein-rich diet); restriction in diet which is largely reinforced by cultural belief systems (*e.g*., oils, fats, nonvegetarian foods etc). 2) Taking rest: This is based on the belief that hard work would lead to weakness in the nerves, which is said to be responsible for panic. Thus, taking rest by sitting under the fan, lying down, etc is said to reduce the chances of panic. 3) Faith healing: About 17% of the sample reported visiting temples, mosques, and involving in other magico-religious healing practices before coming to the hospital.

Overall, the findings of the present study are in accordance with other studies in terms of the outcome and extent of change.[13] The ES also indicates the same—the average ESs for the BI and CBI groups were 0.90 and 1.74, indicating greater magnitude of change. The ES for the CBI group is above the average ES of 0.9 reported for panic control treatment[43] and 0.47 ± 0.3 reported for behavior therapies involving relaxation techniques and supportive techniques without exposure,[44] which may be because of the impact of the multiple components in the CBI group and applied relaxation in the BI group. However, the between group ESs for panic, fear of anxiety, and panic-related cognitions are similar to the earlier studies.[13] The rate of improvement also seems to be similar to the earlier studies.[29]

This study has certain limitations in terms of the small sample size, which limits its generalizability. The outcome was assessed using the self-rating and therapist-rating measures, independent raters were not used; structured long-term follow-up could not be done and hence, maintenance of treatment gains is not known. Semistructured tools were not used to establish diagnosis and co-morbidity. As the treatment methods were explained to the subjects prior to the therapy, it would have had an impact on the expected outcome bias in the patients. Adherence to the treatment program and the patients’ satisfaction of therapy could not be assessed. The number of therapy sessions was different for the two groups, which would have played a role in the improvement. However, the BI group was used only as a control group and patients who did not show adequate improvement were given an option of CBI and medication evaluation, thus taking care of ethical issues.

The strengths of the study are inclusion of drug naïve patients and patients who did not benefit completely with medicines. The treatment package was manual-based and was developed based on existing packages using cognitive behavioral techniques. Care was taken to introduce each technique at a specific point of time. Applied relaxation was tried out successfully as it is found to be effective in panic disorder. Clinical significance of the outcome was assessed along with other appropriate statistical procedures. A control group for comparison was used and the control group received certain common components, increasing the comparability of the two groups.

## CONCLUSION

This study shows that comprehensive treatment is efficacious as an independent treatment modality and it is an effective alternative for patients who are not willing to take pharmacological treatment and who do not benefit completely from pharmacotherapy. CBI is found to be efficacious in handling panic symptoms, cognitions, and behaviors related to panic in a short duration of six weeks. It is also evident that CBI brings about reliable, functional change of a greater magnitude. Future studies may focus on comparative study using different groups with different combinations of techniques to find out the comparative efficacy of different techniques. Influence of the different co-morbid personality states and other co-morbid anxiety disorders on the efficacy of CBT can be examined.
